# Goiter and Its Associated Factors among Adolescent High School Girls at Tach Armachiho District, Northwest Ethiopia: An Institution-Based Cross-Sectional Study

**DOI:** 10.1155/2020/3695952

**Published:** 2020-10-23

**Authors:** Belaynew Mesfin, Bisrat Misganaw, Melkamu Tamir Hunegnaw, Esmael Ali Muhammad

**Affiliations:** ^1^Department of Human Nutrition, Institute of Public Health, University of Gondar, Gondar, Ethiopia; ^2^University of Gondar, College of Medicine and Health Science, Department of Epidemiology and Bio-statistics, Gondar, Ethiopia

## Abstract

**Introduction:**

In Ethiopia, iodine deficiency disorder (IDD) is a major public health problem. The most visible effect of IDD is the appearance of goiters, and 28 million people are affected by goiter. Therefore, this study aimed to assess the prevalence and associated factors of goiter among high school adolescent girls at Tach Armachiho district, Northwest Ethiopia.

**Methods:**

An institution-based cross-sectional study was conducted from October to November 2018. A total of 620 high school adolescent girls were selected using the simple random sampling technique, and structured questionnaires having observational check-list were used for data collection. The presence of goiter was estimated using criteria set by World Health Organization. Iodine content of the salt was estimated by using spot testing kits. Both bivariable and multivariable logistic regressions were used to identify associated factors. The degree of association between independent and dependent variables was assessed by using odds ratio with 95% confidence interval. Those variables having *p* value of less than 0.05 in the multivariable analysis were considered as significant factors for goiter.

**Results:**

A total of 614 adolescent girls gave a complete response. The prevalence of goiter among adolescent girls was 24.1%. In the multivariable logistic regression analysis, age of adolescent girls (adjusted odds ratio (AOR) = 1.57, 95% CI: 1.01–2.46), residence (AOR = 1.91, 95% CI: 1.04–3.51), family history (AOR = 4.96, 95% CI: 3.19, 7.72), low dietary diversity (AOR = 8.39, 95% CI: 4.36–16.14), and medium dietary diversity (AOR = 2.26, 95% CI: 1.36–3.77) were significantly associated with adolescent girls goiter.

**Conclusions:**

Goiter among high school adolescent girls in this study was high. Age of adolescent, residence, family history of goiter, and dietary diversity were statistically significant factors for goiter. Therefore, more emphasis will be given for late adolescent age, having family history of goiter, low dietary diversity, and rural residence to improve the burden of adolescent goiter.

## 1. Introduction

Goiter, which refers to an abnormal enlargement of the thyroid gland, is one of the most common endocrine problems for children and adolescents [1]. Each of the lateral lobes of the thyroid gland is larger than the terminal phalanges of the thumb of a person examined for goiter [2], reflecting a chronic iodine deficiency as a sensitive long-term indicator of the successes of iodine intervention programs [3]. Adolescents are young people aged between 10–19 years [4] who are known to have serious nutritional challenges because of intense physical, psychosocial, and cognitive developments and transition from childhood to adulthood [5]. Interventions during adolescence are used to break the vicious cycle of malnutrition.

The consequences of iodine deficiency are goiter, hypothyroidism, physical and neurophysiologic defects, mental retardation, and brain damage [6]. The educational potential of a nation can be unattainable as iodine deficiency reduces the intelligence quotient (IQ) by 13.5 points due to an inadequate production of the thyroid hormone [7]. The problem is a threat to the productivity of the workforce and a cause of cretinism and mental retardation [8]. To prevent these problems, the Ethiopia government launched the iodized salt fortification, a monitoring and regulatory system in May 2011 [9]. Iodine deficiency is associated with a wide range of physical and mental disorders during the most critical period of development, like adolescent girls and the first trimester of gestation. Furthermore, 30% of all school-age children are mild to severely iodine-deficient, and the deficiency is more prevalent among adolescent girls [10, 11].

Different studies show that the prevalence and associated factors of goiter among adolescent girls varies from country to country. For instance, in Africa, the burden of goiter ranges from 34.5% to 37% on an average; in Ethiopia, about 30% of the adolescent girls develop goiter [12]. The magnitude of goiter also varies from region to region in Ethiopia: in Southern Nations, Nationalities and Peoples region (SNNP) (56.2%), Oromia (42%), Benishangul Gummuz (40%), Amhara (29.1%), and Tigray (21.9%) [7]. The prevalence of goiter is also influenced by factors, such as sex [13], educational status and age [14–16], place of birth and family income [17], residence [18], consumption of iodized salt [17], knowledge, dietary intake, drinking unprotected water, eating goitrogenic foods, and family history [18].

Although the Government of Ethiopia has increased its efforts to prevent goiter, its magnitude among adolescent girls is still high, and factors vary from place to place. Therefore, this study aimed to assess the magnitude of goiter and associated factors among adolescent girls in Tach Armachiho district.

## 2. Methods and Materials

### 2.1. Study Setting and Sample Size

An institution-based cross-sectional study was conducted from October to November 2018 among adolescent high school girls at Tach Armachiho district, Northwest Ethiopia, located 854 km from Addis Ababa, the capital of Ethiopia. The district has 38 kebeles (the smallest administrative units). Its altitude is 1050 to 1800 ms above sea level; 42% of its area is mountainous, with a temperature of 25–42 degree Celsius. According to the 2018 report, the district had four high schools with 1,972 adolescents. One district hospital and nine health centers provide health services.

The single population proportion formula was used to determine the sample size by considering 37.6% previous prevalence of goiter [14], 95% confidence interval, 4% margin of error, and 10% none response rate, which yielded 620. The sample size was distributed proportionally to four high schools based on the number of students in each school. The simple random sampling technique was used to select study participants.

### 2.2. Inclusion and Exclusion Criteria

Adolescent girls who were learning in high school during data collection were included in the study, and adolescent girls who suffered from serious illness during the period of data collection were excluded from the study.

### 2.3. Assessment of Goiter and Salt Iodine Content

For the general data collection, eight nurses, two general practitioners, and two public health experts participated as supervisors, after they were given two-day training by the principal investigator. In order to maintain data quality, a pretest was administered on 5% of the sample in Tsegede district out of the study area. Onsite supervision was performed, and each copy of the questionnaire was checked for completeness and accuracy before data entry.

Physical examination of a thyroid gland was performed by two skilled general practitioners (GPs), using WHO/ICCIDD/UNICEF clinical criteria. Accordingly, goiter was defined as grade 0 if no palpable mass in the neck was detected, grade 1 if there was a mass in the neck consistent with palpable enlarged thyroid, but not visible when the neck was in the normal position, whereas grade 2 if there was a swelling in the neck that was visible when the neck was in a normal position and is consistent with an enlarged thyroid when the neck is palpated (palpable and visible). Lastly, the child was deemed as having goiter when he/she had goiter of grade 1 or 2 [19].

Iodine level of salt was determined by a rapid test kit (ARCHIV MANNAR no. 107397). The kit was used in houses of all participants; the interviewer asked households to provide a teaspoon of salt used for cooking and added two drops of test solution to determine the iodine content in the salt. It was filled in a small cup and spreaded flat, two drops of test solution were added on the surface of salt by piercing the white ampule, the color of salt was compared with a color chart within one minute, and the iodine concentration was determined (intense color). If no color change appeared on the salt after one minute, up to five drops of recheck solution were added in red ampule on a fresh sample, two drops of test solution were added on the same spot, and the color was compared with the color chart. The iodine content was determined, if it is 0 parts per million (no iodine), <15 ppm (light blue), ≥15 ppm (deep blue) [16, 20].

### 2.4. Assessment of Household Wealth Status

Household's wealth index, adopted from EDHS 2011, was determined using principal component analysis (PCA) by considering the household assets, such as quantity of cereal products, type of house, livestock, and agricultural land ownership. First, variables were coded between 0 and 1 and then entered and analyzed using PCA. Those variables having a communality value of greater than 0.5 were used to produce factor scores. Finally, the factor scores were summed and ranked into tertiles as poor, medium, and rich [21].

### 2.5. Data Processing and Analysis

The collected data were entered by using Epi Info 7 and transferred to SPSS version 20 for further analysis. Data cleaning was performed to check for accuracy, consistency, and missed values. Frequencies, proportions, and summary statistics were used to describe the variables.

The result was presented using tables, and statistical associations were assessed by using bivariable and multivariable logistic regressions to obtain crude and adjusted odds ratios with a 95% confidence interval. Variables with less than or equal to 0.2 *p* values in the bivariable analysis were entered into the multivariable analysis to control the possible effects of confounders. Variables with *p* value <0.05 in the multivariable analysis were considered as statistically significant for adolescent goiter.

## 3. Results

### 3.1. Sociodemographic and Environment-Related Characteristics

A total of 614 adolescent girls participated with a response rate of 99.03%. The mean age of the participants was 16.78 years with SD ± 1.66. A majority of the participants, 380 (61.9%), were in the age group of 16–19 years; 588 (95.8%) were Orthodox Christian; 290 (47.2%) were grade nine completed; 435 (70.8%) were rural dwellers, and 214 (34.9%) had low economic status; 510 (83.1%) of the fathers were farmers; 517 (84.2%) of the mothers/care givers were housewives, and 466 (75.9%) were unable to read and write (Table 1).

### 3.2. Utilization of Iodized Salt and Dietary Practice-Related Characteristics

In this study, 448 (73%) households used adequate iodized salt (≥15 ppm). Of the participants, 580 (84.4%) used iodized salt during food preparation, 259 (50%) added it at the end of cooking, and 422 (68.7%) households stored salt without exposing it to direct sunlight. One hundred and sixty (26%) of the participants had a family history of goiter, and 448 (71.3%) had adequate dietary diversity score in the last 24 hours (Table 2).

### 3.3. Prevalence of Goiter

The overall prevalence of goiter among adolescent girls was 24.1% (CI: 95%, 20.6–27.5); 13.2% and 10.9% of the goiters were grade 1 and grade 2, respectively.

### 3.4. Factors Associated with Goiter

The result of the multivariable analysis revealed that age, residence, family history of goiter, and DDS were significantly associated with goiter. Accordingly, the odds of goiter were 1.57 times more likely among late than early adolescent girls (AOR: 1.57; 95% CI: 1.01, 2.46). Likewise, the odds of getting goiter were 2 times higher among participants who lived in rural areas than in urban settings (AOR: 1.91; 95% CI: 1.04, 3.51). Adolescent girls who had a family history of goiter were more likely to develop the problem than their counterparts (AOR: 4.96; 95% CI: 3.19, 7.72). Similarly, higher odds of goiter were noted among adolescent girls who consumed inadequately diversified diets than their counterparts (AOR: 8.39; 95% CI: 4.36, 16.14) (Table 3).

## 4. Discussion

The prevalence of goiter among adolescent girls school at 24.1% (20.0–29.9%) posed a moderate public health challenge to the study area [22]. This finding was lower than those of studies done in two zones of Ethiopia, such as Metekel (39.4%) [23] and Wolaita (60.9%) [18], Bangladesh (44%) [13] and Rawalpindi, and Pakistan (57.1%) [24]. The possible justification for this disparity might be variations in topography and dietary habits; for instance, in Wolaita, Ethiopia, the community had a dietary history of frequent and high (81.3%) consumption of cassava and poor utilization of iodized salt. In addition, the variations might be due to differences in study settings and periods. However, the result of our study was higher than those studies done in Charsadda, Pakistan (11.5%) [25], and Uttarakhand, India (15.9%) [26]. This difference might be due to variations in the topographies of the study areas. This study was done in a mountainous (high altitude) area that resulted in poor soil conservation over a long period of time and contributed to leaching away of the iodine-rich soil layer, exposing the iodine-poor layer beneath.

Late adolescent (16–19 years) girls were more likely to be affected by goiter than early age groups. This finding was consistent with the results of studies done in Hawassa, Goba, and Robe, Ethiopia [14, 15, 17], respectively. This might be due to the fact that iodine requirement increases with age, and older children had prolonged exposure to iodine-deficient environment. Besides, thyroid size correlates with body surface area and increases with age, making an enlarged thyroid more visible and palpable in the age of adolescents [18, 23].

In our study, rural dweller adolescent girls were more likely to have goiter compared with urban residents. The result was consistent with the findings of Gamogofa [27] and Lay Armachiho zones, Ethiopia [28]. The possible reason might be difference in knowledge about the causes and preventions of goiter and inadequate diversified food. It could also be due to variations in iodized salt storage area that affects the content of iodine because of its volatile nature, and the commonly stable maize and millet diet of the rural community has poor iodine content.

Adolescent girls who had a family history of goiter were more likely to develop goiter than their counterparts. This finding was supported by the result obtained in Lay Armachiho and Gamogofa, Ethiopia [28, 29], Brazil [30], and Germany [31]. The possible reason might be that family clustering of goiter has been common historically, and a complex “multifactorial” interactions of genetic and shared environmental factors lead to higher rates of goiter among people [32].

Dietary diversity of adolescent girls was significantly associated with goiter. Poor DDS among adolescent girls was almost 8 times more likely to develop goiter compared to adolescent girls without the problem. The result was supported by studies done in the Amhara regional state, Adama city and Bale, Ethiopia [33–35], respectively. The possible justification for this could be that when adolescent girls have no access to diversified foods, the probability of getting iodine is low. One of the iodine deficiency disorder strategies is adequate dietary intake. Communities with cereal-based monotonous dietary habits suffer from iodine and other micronutrient deficiencies, such as vitamin A and iron [21].

## 5. Conclusion

The overall prevalence of goiter among adolescent girls was a moderate public health problem. Age, residence, family history, and DDS were significantly associated with goiter. Therefore, due emphases should be given to late adolescents by counseling about diversified diets and the causes and prevention of goiter.

## Figures and Tables

**Table 1:** Sociodemographic and environmental-related characteristics of adolescent girls in Tach Armachiho district, 2018 (*N* = 614).

Variable	Category	Frequency	Percentage
Age	10–15	234	38.01
	16–19	380	61.90

Religion	Orthodox	588	95.8
	Catholic	3	0.5
	Muslim	23	3.7

Grade level	Grade 9	290	47.2
	Grade 10	143	23.3
	Grade 11	101	16.4
	Grade 12	80	13.0

Residence	Urban	179	29.2
	Rural	435	70.8

Fathers occupation	Government employee	47	7.7
	Farmer	510	83.1
	Daily labor	12	2.0
	Merchant	45	7.3

Mothers occupation	Government employee	10	1.6
	Farmer	517	84.2
	Daily labor	5	0.8
	Merchants	35	5.7
	Nongovernment employee	47	7.7

Maternal education	Illiterate	466	75.9
	Informal education	95	15.5
	Primary school (1–8) grade	34	5.5
	Secondary school and above	19	3.1

Family size	<5	367	59.8
	>5	247	40.2

Wealth status	Poor	214	34.9
	Medium	211	34.4
	Rich	189	30.8

Source of drinking water	Tap water	252	41.0
	Public tap	153	24.9
	Protected well	177	28.8
	Unprotected well	32	5.2

Practice water safe to drink	Boiled	78	12.7
	Wuhagar/chlorine added	457	74.4
	Strained through cloth	7	1.1
	Sunlight with bottle	38	6.2
	Others^*∗∗*^	34	5.5

Community gardening	Yes	277	45.1
	No	337	54.9

Community gardening (*n* = 277)	Selling all	39	14.1
	Selling some of	132	47.7
	All used by the family	92	33.2
	Others	14	5.1

^*∗∗*^Add sand and settle and sediment the dirty pieces.

**Table 2:** Utilization of iodized salt and dietary practice-related characteristics of adolescent girls in Tach Armachiho district, Northwest Ethiopia, 2018 (*N* = 614).

Variable	Category	Frequency	Percentage
Use iodized salt during cooking “wott”	Yes	518	84.4
	No	96	15.6

Salt added during cooking (*n* = 518)	Early	106	20.5
	Middle of cooking	88	17
	End of cooking	259	50
	After cooking	65	12.5

Salt storage in the house	By opened parcel	77	12.5
	By closed parcel	537	87.5

Salt exposure to sunlight	Yes	192	31.3
	No	422	68.7

Storage of salt relation to fire	Near to fire	224	36.5
	Away from fire	390	63.5

Current salt iodine level	“0” PPM	36	5.9
	1–15 PPM	96	15.6
	>15 PPM	448	73
	No salt got in house	34	5.5

Family history of goiter	Yes	161	26.2
	No	453	73.8

Get treatment (*n* = 90)	Traditional healthier	37	41.1
	Health institution	53	58.9

Dietary diversity score (DDS)	Poor	56	9.1
	Moderate	120	19.5
	Good	438	71.3

**Table 3:** Factors associated with goiter among adolescent girls in Tach Armachiho district, Northwest Ethiopia, 2018.

Variable	Category	Goiter		COR (95% CI)	AOR (95% CI)
		Yes	No		

Age	10–15	45	189	1	1
	16–19	103	277	1.56 (1.05, 2.32)	**1.57 (1.01, 2.46)** ^*∗*^

Maternal education	Illiterate	108	358	0.42 (0.16, 1.06)	0.31 (0.10, 0.96)
	Informal education	22	73	0.41 (0.15, 1.16)	0.37 (0.11, 1.26)
	Primary school	10	24	0.57 (0.18, 1.9)	0.51 (0.13, 1.98)
	Secondary and above	8	11	1	1

Source of drinking water	Tap water	54	198	1	1
	Public tap	40	113	1.29 (0.81, 2.08)	0.99 (0.53, 1.85)
	Protected wall	42	135	1.14 (0.72, 1.80)	0.69 (0.38, 1.28)
	Unprotected wall	12	20	2.20 (1.01, 4.78)	1.18 (0.45, 3.12)

Family size	<5	79	288	1	1
	>5	69	178	1.41 (0.97, 2.05)	1.11 (0.72, 1.70)

Residence	Rural	117	318	1.76 (1.13, 2.73)	**1.91 (1.04, 3.51)** ^*∗*^
	Urban	31	148	1	1

Family history	No family history	71	382	1	1
	Had family history	77	84	4.93 (3.31, 7.36)	**4.96 (3.19, 7.72)** ^*∗*^

Salt storage	Open parcel	24	53	1.5 (0.89, 2.54)	1.19 (0.65, 2.16)
	Close parcel	124	413	1	1

Salt exposure to sun light	Yes	56	136	1.48 (1.0, 2.2)	1.33 (0.83, 2.14)
	No	92	330	1	1

Salt storage site	Near to fire	65	159	1.51 (1.04, 2.20)	1.52 (0.97, 2.38)
	Away from fire	83	307	1	1

Dietary diversity (DDS)	Poor	33	23	6.73 (3.74, 12.09)	**8.39 (4.36, 16.14)** ^*∗*^
	Medium	38	82	2.17 (1.38, 3.43)	**2.26 (1.36, 3.77)** ^*∗*^
	Good	77	361	1	1

^*∗*^Significant at *p* value less than 0.05.

## Data Availability

Full data set and materials pertaining to this study can be obtained from the corresponding author upon reasonable request.

## Abbreviations

AOR: Adjusted odds ratio
BCC: Behavioral change and communication
CI: Confidence interval
COR: Crude odds ratio
DDS: Dietary diversity score
GP: General practitioner
HH: Household
ICCIDD: International Council for the Control of Iodine Deficiency Disorders
IDD: Iodine deficiency disorder
IQ: Intelligence quotient
PPM: Parts per million
SPSS: Statistical Package for the Social Sciences
SNNP: Southern Nations, Nationalities and Peoples
TGR: Total goiter rate
TSH: Thyroid stimulating hormone
UNICEF: United Nations International Cultural and Educational Foundation
WHO: World Health Organization.

## Additional Points

The limitation of this study might be observer bias in grading goiter and inability to get the appropriate of ultrasound findings.

## References

[1] Roti E., Gardini E., D’amato L. (1986). Goiter size and thyroid function in an endemic goiter area in northern Italy. *The Journal of Clinical Endocrinology & Metabolism*.

[2] World Health Organization (2014). *Goitre as a Determinant of the Prevalence and Severity of Iodine Deficiency Disorders in Populations*.

[3] De Benoist B. (2004). *Iodine Status Worldwide. WHO Global Database on Iodine Deficiency*.

[4] World Health Organization (2005). *Nutrition in Adolescence: Issues and Challenges for the Health Sector: Issues in Adolescent Health and Development*.

[5] Das J. K., Salam R. A., Thornburg K. L. (2017). Nutrition in adolescents: physiology, metabolism, and nutritional needs. *Annals of the New York Academy of Sciences*.

[6] Kidane T., Woldegebriel A. (2006). Prevalence of Iodine deficiency disorder in a highland district in Tigray. *Ethiopian Journal of Health Development*.

[7] Yadav K., Pandav C. (2018). National iodine deficiency disorders control programme: current status & future strategy. *Indian Journal of Medical Research*.

[8] Bogale A., Abebe Y, Stoecker B. J, Abuye C, Ketema K, Hambidge K. M (2009). Iodine status and cognitive function of women and their five year-old children in rural Sidama, southern Ethiopia. *East African Journal of Public Health*.

[9] Gebremariam H. G., Yesuf M. E., Koye D. N. (2013). Availability of adequately iodized salt at household level and associated factors in Gondar town, Northwest Ethiopia. *International Scholarly Research Notices Public Health*.

[10] Andersson M., Karumbunathan V., Zimmermann M. B. (2012). Global iodine status in 2011 and trends over the past decade. *The Journal of Nutrition*.

[11] Vanderpump M. P. (2011). The epidemiology of thyroid disease. *British Medical Bulletin*.

[12] Andersson M., de Benoist B., Rogers L. (2010). Epidemiology of iodine deficiency: salt iodisation and iodine status. *Best Practice & Research Clinical Endocrinology & Metabolism*.

[13] Ara G., Melse-Boon A., Roy S. K. (2010). Sub-clinical iodine deficiency still prevalent in bangladeshi adolescent girls and pregnant women. *Asian Journal of Clinical Nutrition*.

[14] Mesele M., Degu G., Gebrehiwot H. (2014). Prevalence and associated factors of goiter among rural children aged 6-12 years old in Northwest Ethiopia, cross-sectional study. *BioMed Central Public Health*.

[15] Girma M. (2012). Iodine deficiency in primary school children and knowledge of iodine deficiency and iodized salt among caretakers in Hawassa Town: southern Ethiopia. *Ethiopian Journal of Health Development*.

[16] Gebretsadikan T. M., Troen A. M. (2016). Progress and challenges in eliminating iodine deficiency in Ethiopia: a systematic review. *BioMed Central Nutrition*.

[17] Enyew H. D., Zemedkun K. G., Dagnaw A. M. (2015). Prevalence of goiter and associated factors among primary school children aged 6–12 Years old in GobaTown, South east, Ethiopia. *International Journal of Food Sciences and Nutrition*.

[18] Workie S. B. (2017). Assessing the status of iodine deficiency disorder (IDD) and associated factors in Wolaita and Dawro Zones School Adolescents, southern Ethiopia. *BioMed Central Research Notes*.

[19] Kamath R. (2009). Prevalence of Goiter in rural area of Belgaum district, Karnataka. *Indian Journal of Community Medicine: Official Publication of Indian Association of Preventive & Social Medicine*.

[20] Hawas S. (2016). Proper utilization of adequatly iodized salt at house hold level and associated factores in Asella Town Arsi Zone Ethiopia: a community based cross sectional study. *Journal of Food Processing and Technology*.

[21] Abebe Z., Gebeye E., Tariku A. (2017). Poor dietary diversity, wealth status and use of un-iodized salt are associated with goiter among school children: a cross-sectional study in Ethiopia. *BioMed Central Public Health*.

[22] World Health Organization, Disorders I.C.f.C.o.I.D. (1994). *Indicators for Assessing Iodine Deficiency Disorders and Their Control through Salt Iodization*.

[23] Kibatu G., Nibret E., Gedefaw M. (2014). The status of iodine nutrition and iodine deficiency disorders among school children in Metekel Zone, northwest Ethiopia. *Ethiopian Journal of Health Sciences*.

[24] Shahid A. (2009). Assessment of nutritional status of adolescent college girls at Rawalpindi. *Annals of King Edward Medical University,*.

[25] Jahangir M. (2015). Prevalence of Goiter and iodine nutritional status in school age children of district karak, khyber pakhtunkhwa, Pakistan. *Acta Endocrinologica*.

[26] Kapil U., Pandey R. M, Prakash S, Kabra M, Sareen N, Bhadoria A. S (2014). Assessment of iodine deficiency in school age children in Nainital District, Uttarakhand State. *Asia Pacific Journal of Clinical Nutrition*.

[27] Kebede D. L. (2015). Predictors of goiter among school children in southwest Ethiopia case control study. *Nutrition & Food Sciences*.

[28] Mesele M., Degu G., Gebrehiwot H. (2014). Prevalence and associated factors of goiter among rural children aged 6-12 years old in Northwest Ethiopia, cross-sectional study. *BioMed Central Public Health*.

[29] Abuye C., Omwega A. M., Imungi J. K. (1999). Familial tendency and dietary association of goitre in Gamo-Gofa, Ethiopia. *East African Medical Journal*.

[30] Afm D.v.f.m. (2011). Sex Effect on the familial agregation of endemic goiter. *Braziljgenetics*.

[31] Wicht J., Singer J., Paschke R. (2008). Genetic predisposition for goiters analysed by a case control study in Germeny. *Endocrine Abstracts*.

[32] Böttcher Y., Eszlinger M., Tönjes A., Paschke R. (2005). The genetics of euthyroid familial goiter. *Trends in Endocrinology & Metabolism*.

[33] Yasin A., Mohammed T. B. T. (2015). Nutritional status and associated risk factors among adolescent girls in agarfa high school, Bale zone, Oromia region, South east Ethiopia. *International Journal of Nutrition and Food Sciences*.

[34] Roba K. T., Abdo M., Wakayo T. (2016). Nutritional status and its associated factors among school adolescent girls in Adama city, Central Ethiopia. *Journal of Nutrition & Food Sciences*.

[35] Wassie M. M., Gete A. A., Yesuf M. E. (2015). Predictors of nutritional status of Ethiopian adolescent girls: a community based cross sectional study. *Bio Nutrition*.

